# Exploring the Central Mechanisms of Botulinum Toxin in Parkinson’s Disease: A Systematic Review from Animal Models to Human Evidence

**DOI:** 10.3390/toxins16010009

**Published:** 2023-12-23

**Authors:** Carolina Cutrona, Francesco Marchet, Matteo Costanzo, Maria Ilenia De Bartolo, Giorgio Leodori, Gina Ferrazzano, Antonella Conte, Giovanni Fabbrini, Alfredo Berardelli, Daniele Belvisi

**Affiliations:** 1 Department of Human Neurosciences, Sapienza University of Rome, Viale dell’Università 30, 00185 Rome, Italy; carolina.cutrona@uniroma1.it (C.C.); francesco.marchet@uniroma1.it (F.M.); mariailenia.debartolo@uniroma1.it (M.I.D.B.); giorgio.leodori@uniroma1.it (G.L.); gina.ferrazzano@uniroma1.it (G.F.); antonella.conte@uniroma1.it (A.C.); giovanni.fabbrini@uniroma1.it (G.F.); alfredo.berardelli@uniroma1.it (A.B.); 2 Department of Neuroscience, Istituto Superiore di Sanità, Viale Regina Elena 299, 00161 Rome, Italy; matteo.costanzo@uniroma1.it; 3 IRCSS Neuromed, Via Atinense 18, 86077 Pozzilli, Italy

**Keywords:** botulinum toxin, Parkinson’s disease, central nervous system, animal models

## Abstract

Botulinum toxin (BoNT) is an effective and safe therapy for the symptomatic treatment of several neurological disturbances. An important line of research has provided numerous pieces of evidence about the mechanisms of action of BoNT in the central nervous system, especially in the context of dystonia and spasticity. However, only a few studies focused on the possible central effects of BoNT in Parkinson’s disease (PD). We performed a systematic review to describe and discuss the evidence from studies focused on possible central effects of BoNT in PD animal models and PD patients. To this aim, a literature search in PubMed and SCOPUS was performed in May 2023. The records were screened according to title and abstract by two independent reviewers and relevant articles were selected for full-text review. Most of the papers highlighted by our review report that the intrastriatal administration of BoNT, through local anticholinergic action and the remodulation of striatal compensatory mechanisms secondary to dopaminergic denervation, induces an improvement in motor and non-motor symptoms in the absence of neuronal loss in animal models of PD. In human subjects, the data are scarce: a single neurophysiological study in tremulous PD patients found that the change in tremor severity after peripheral BoNT administration was associated with improved sensory–motor integration and intracortical inhibition measures. Further clinical, neurophysiological, and neuroimaging studies are necessary to clarify the possible central effects of BoNT in PD.

## 1. Introduction

Botulinum toxin (BoNT) is an approved, safe, and effective drug for the symptomatic treatment of several neurological and non-neurological conditions. The main effect of BoNT therapy, consisting of a transient chemical paralysis of the treated muscles, stems from the blockage of the release of acetylcholine (Ach) from the motor axons of the neuromuscular junction [1]. After the injection, through the binding with proteins, including synaptotagmin and synaptic vesicle protein 2, BoNT is internalised in the presynaptic terminal where it cleaves and deactivates the SNARE proteins (SNAP 25, VAMP, syntaxin) that are essential for the fusion of vesicles with the synaptic membrane and the release of Ach into the synaptic cleft [2]. BoNT is commonly used in the treatment of many neurological disorders, including dystonia and spasticity, among others [3,4]. In recent years, however, growing attention has been paid to the possible application of BoNT to treat motor and non-motor symptoms in Parkinson’s disease (PD) [5,6]. The appeal of BoNT stems from its targeted action, the absence of widespread side effects, and its compatibility with dopaminergic treatments, offering a promising avenue for treating symptoms that traditionally relied on systemically active medications. The symptoms in PD that are amenable to treatment with BoNT include motor symptoms such as tremors, focal dystonia, and dyskinetic movements, as well as non-motor symptoms, including sialorrhea, dysphagia, gastroparesis, constipation, bladder hyperactivity, and sweating dysfunction [6,7]. The evidence derived from studies conducted on patients suffering from spasticity and dystonia suggests that BoNT acts not only on the neuromuscular junction but also at the level of intrafusal muscle fibres, neuromuscular spindles, the spinal cord, and suprasegmental structures [8,9,10,11,12,13,14,15,16,17,18,19,20]. The action of BoNT at these levels could explain its wide and long-lasting effects [21,22]. Unlike dystonia and spasticity, only a few studies have provided evidence regarding potential central effects of BoNT in PD.

In this systematic review, we aimed to evaluate the studies conducted so far on both animal models and patients with PD that focused on examining and clarifying the possible central effects of BoNT in PD. 

## 2. Methods

### 2.1. Search Strategy

**Figure 1:**
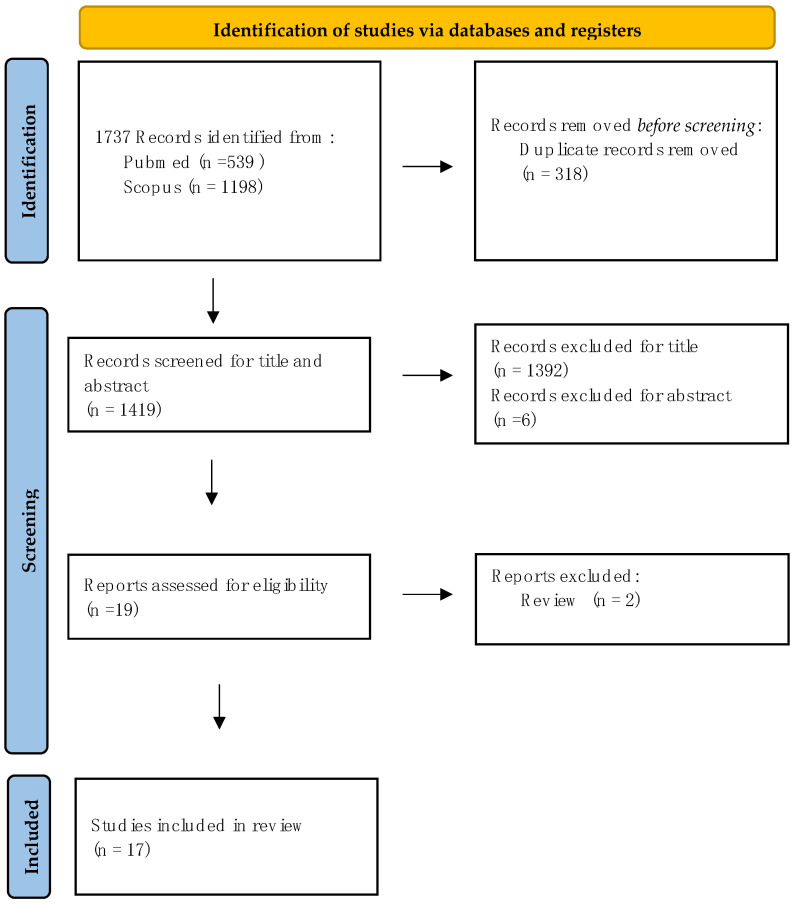
Preferred Reporting Items for Systematic Reviews and Meta-Analysis (PRISMA) on central effect of botulinum toxin (BoNT)-A in PD animal models and patients. Reprinted with permission from reference [24].

This systematic review was performed following the Preferred Reporting Items for Systematic Reviews and Meta-Analysis (PRISMA) flow diagram (https://www.bmj.com/content/339/bmj.b2700, accessed on 4 May 2023). The PRISMA flow diagram is presented in Figure 1 [23].

Literature searches in PubMed and SCOPUS were performed in May 2023 using the following search string: ((Botulinum toxin [Title/Abstract]) OR BoNT [Title/Abstract]) AND Parkinson’s disease [Title/Abstract] OR PD [Title/Abstract]. The searches were limited to publications in English. All articles published before May 2023 were included. All search results were aggregated in Excel for Windows, and duplicates were discarded, so only unique references were retained. This review was not registered.

### 2.2. Study Selection

The records were screened according to their title and abstract by two independent reviewers (CC and FM). The relevant articles were selected for full-text review. Additionally, the studies where the eligibility remained undetermined based solely on the title or abstract were also considered for a full-text review.

Eligible studies for inclusion were as follows:

(I) Firstly, studies exploring PD animal models where molecular, motor behavioural, and non-motor changes after central nervous system (CNS) injection of BoNT were assessed.
(II) Secondly, studies focusing on PD human subjects that evaluated the central effect of BoNT treatment through clinical, neuroradiological, and neurophysiological assessments.

In instances where disagreement arose between the reviewers, debates were conducted until a unanimous decision was achieved regarding the inclusion of the study. This systematic review was open to including both longitudinal and cross-sectional studies. We imposed no restrictions regarding the age of participants or the duration of their disease, as our goal was inclusivity. As we aimed to produce a comprehensive review of any evidence of the effect of BoNT in the CNS, no study quality threshold was set.

## 3. Results

### 3.1. Evidence from Animal Models

Our systematic search identified 17 longitudinal studies performed in animal models. Most studies were performed in the 6-hydroxydopamine (6-OHDA) hemi-PD model. Only one study utilised the 1-methyl-4-phenyl-1,2,3,6-tetrahydropyridine (MPTP) mouse model of PD [25] (see Table 1).

6-OHDA is a molecule similar to dopamine that, when it binds to the dopamine transporter molecule, can reach neurons, causing strong oxidative stress in the cytosol and mitochondria. The injection of 6-OHDA into the median forebrain bundle (MFB) induces almost total dopaminergic denervation in the substantia nigra pars compacta (SNPc) in 2–3 days [43]. In most of the studies included in our search, the 6-OHDA toxin was injected into the right MFB, obtaining a hemi-parkinsonian model. MPTP is a compound that is able to cause a selective degeneration of the SNPc after systemic administration and has been widely used in the last 30 years to obtain a PD animal model. In the only study we included in our selected studies that adopted this approach, the protocol consisted of an intraperitoneal injection four times per day for 7 days in C57BL/6 mice [25].

#### 3.1.1. Molecular Evidence

We identified eleven studies that employed immunohistochemistry, immunofluorescence, autoradiography, positron emission tomography (PET), and functional magnetic resonance imaging (fMRI) techniques to examine alterations in synaptic transmission and receptor systems after intracerebral administration of BoNT within the striatum. 

In the following sections, we will discuss separately the evidence regarding the effects of BoNT on cholinergic, dopaminergic, GABAergic, serotonergic, noradrenergic, and glutamatergic pathways in hemi-PD animal models. 

##### BoNT Effects on Cholinergic System

SNAP-25 represents one of the pivotal targets through which BoNT interrupts cholinergic transmission at the presynaptic level [44,45]. A study employing immunostaining against cleaved SNAP-25 in rats described that, after BoNT intrastriatal injection, there is an increase in cleaved SNAP-25 immunostaining limited to the ipsilateral striatum, and motor and somatosensory cortices, sparing the contralateral hemisphere and interhemispheric connection structures [46]. In addition, the impact of BoNT was subtype-dependent, with BoNT-A2 exhibiting more pronounced effects [27].

Three studies investigated the impact of intrastriatal BoNT injections on local cholinergic transmission in animal models of PD. Two investigations highlighted the morphological modifications indicative of Ach accumulation at presynaptic terminals [26,36]. In a 6-OHDA PD rat model, Wree et al. (2011) observed the development of varicosities, which were immunohistochemically positive for choline acetyltransferase (ChAT), along striatal neurons on the ipsilateral side of the intrastriatal BoNT injection [26]. Analogous results were reported by Hawlitschka et al. in a study conducted on C57BL/6 mouse models [28]. Importantly, both studies concluded that BoNT does not affect the count of ChAT-positive interneurons, implying it does not exert toxic effects on cholinergic neurons [26,28]. 

In a nine-month longitudinal study, Mann et al. used in vitro receptor autoradiography to delineate the temporal trajectory of BoNT-induced alterations in the densities of various muscarinic and nicotinic receptor subtypes within the striatum of 6-OHDA rats. The investigation uncovered distinct temporal patterns of receptor density alterations after the intrastriatal injection of BoNT. Specifically, the density of M1 receptors initially diminished three weeks post-intervention, followed by a progressive recovery over the ensuing nine months. Conversely, densities of M2 and M4 receptors, initially stable, showed a reduction over the subsequent nine months without apparent recovery. The M3 receptors consistently manifested a 10% reduction in density, which persisted throughout the study. Lastly, nicotinic receptors, especially the α4 and β2 subtypes, exhibited a substantial 50–60% decrease post-injection, with no indication of subsequent recovery [29]. 

In the studies mentioned above, potential correlations between BoNT-induced ultrastructural changes and motor behavioural measures were not explored. 

Overall, the identified morphological changes and modulation of receptor density shed light on the intricate, time-dependent, and eventually network-specific impacts of BoNT, underscoring its non-toxic impact on cholinergic neurons and suggesting potential therapeutic implications in PD models. 

##### BoNT Effects on Dopaminergic System

In our systematic review, we identified five studies that focused on the effect of intrastriatal BoNT injections on dopaminergic neurotransmission.

The dopaminergic denervation, reproduced experimentally in 6-OHDA rats and MPTP mice, triggers compensatory mechanisms, including increased expression of striatal dopaminergic receptors D1R and D2R, increased interhemispheric differences in dopamine receptor expression, and an increased acetylcholine/dopamine ratio in lysates of whole brain tissue [25].

By using immunohistochemistry and ELISA on lysates of the whole brain, Ham et al. observed that intrastriatal BoNT injections induce a recovery of dopamine levels and an increase in the expression of tyrosine hydroxylase-positive neurons in the SNpc, which are both reduced by 6-OHDA- or MPTP-induced lesions. 

The BoNT-induced effect on the compensatory modification of dopaminergic receptor expression has been studied both in vitro and in vivo, using autoradiography and positron emission tomography (PET) in animal models of PD [32,33]. Three longitudinal studies showed a significant reduction in ipsilateral D1R expression and a reduction in interhemispheric differences in striatal binding of D2R in 6-OHDA rats treated with intrastriatal BoNT compared to sham-treated animals [26,28,30].

A correlation between motor behavioural measures and striatal changes in dopamine receptor expression has also been reported. In 6-OHDA rats, Wedekind et al. found a positive correlation between asymmetry in forelimb use, evaluated through the cylinder test (see below), and the inter-striatal difference in D2R binding [31]. In a similar animal model, Mann et al. described a significant positive correlation between the difference in D2R/D3R availability in the right/left striatum and an increase in apomorphine-induced rotational behaviour (see below) [33].

Overall, these studies might suggest that striatal BoNT injections, by modulating compensatory mechanisms arising from dopaminergic denervation, can improve motor performance in animal models of PD. 

In our review, we only identified one study that focused on the assessment of D2/D3 receptors at the level of the olfactory bulb in 6-OHDA rats treated with intrastriatal BoNT using PET/TC [34]. The authors showed that the administration of BoNT into the striatum increased both D2R and D3R availability within the olfactory bulb, accompanied by improved olfactory performance [34]. The evidence coming from this study confirms that dopamine is a key transmitter for processing olfactory information in the olfactory bulb. The authors hypothesised that the increase in receptor availability in the OB after ipsilateral striatal BoNT injection parallels olfactory performance and occurs through indirect connections between the striatum and olfactory bulb via the dorsal raphe nucleus or the amygdala nuclear complex [34]. 

##### BoNT Effects on Glutamatergic and GABAergic Systems

We only identified one study assessing BoNT’s impact on the glutamatergic and GABAergic neurotransmitter systems. BoNT injections were administered into the entopeduncular nucleus (EPN) of 6-OHDA rats, a region analogous to the human globus pallidus internus (GPi), in an attempt to target presynaptic glutamatergic inputs from the subthalamic nucleus (STN) [35]. An immunohistochemical analysis using markers specific to glutamatergic (vesicular glutamate transporter 2- vGlut2) and GABAergic (GAD-67) terminals was performed. While no significant modifications were observed in GABAergic immunoreactivity, a significant reduction was detected in glutamatergic immunoreactivity. Even though the chronological trajectory between BoNT-induced immunohistochemical and motor behavioural changes was similar, the presence of potential correlations between these measures was not investigated [35]. 

Overall, these findings indicate that BoNT might be able to modulate glutamatergic systems, leaving GABAergic systems unaffected. These observations might suggest the possibility of a neurochemical modulation of the STN through the local injection of BoNT, with a therapeutic target similar to what has been observed in PD patients treated with deep brain stimulation (DBS) of the STN.

##### BoNT Effects on Serotoninergic and Noradrenergic Systems

Our search identified only one study that focused on the effect of intrastriatal BoNT injections on the serotoninergic and noradrenergic systems. In this study, Mann et al. employed quantitative in vitro receptor autoradiography in the 6-OHDA PD animal model to measure BoNT-induced changes in alpha 1, alpha2, and 5HT2A receptors at the striatal level. The mean density and the relative interhemispheric right–left difference were assessed for each receptor [32]. 

No changes were observed for alpha1 and alpha 2 receptors at the striatal level after the BoNT injection. Conversely, there was an initial 50% reduction in the mean density of the 5HT2A receptor in 6-OHDA PD rats, which underwent a further reduction following the BoNT injection. Finally, no significant correlation was reported between these receptors’ alterations and motor improvements, as assessed by the rotational test. 

Overall, these observations suggest that the noradrenergic and serotonergic systems do not represent preferential targets of BoNT at the striatal level in animal PD models [32]. 

#### 3.1.2. Evidence for BoNT Effects on Motor Behaviour

We identified eleven sham-controlled longitudinal studies investigating motor behaviour changes in PD animal models following the intracerebral injection of BoNT. While the majority of studies administered BoNT into the striatum, two studies injected BoNT into the EPN, which is equivalent to the human GPi [25,26,27,31,33,35,36,37,38,40]. The studies also varied in terms of the dosage of BoNT used and whether the injection was ipsilateral or contralateral to the lesioned side.

Beyond the differences in the injection methodologies employed, the outcome measures used across these studies were also quite heterogeneous. Indeed, various motor behaviour measures were employed, including analysis of drug-induced motor behaviours and performance evaluations conducted through validated motor tasks. The type of motor tasks that were used are shown in Table 2. 

Drug-induced motor behaviours were investigated using apomorphine and amphetamine, which induce rotational behaviours in animals either opposite (apomorphine) or on the same side (amphetamine) of the 6-OHDA-induced lesion. Apomorphine, a dopamine agonist, binds more to the supersensitive dopamine receptors (DRs) on the lesioned side than the contralateral normal DRs and induces an anticlockwise rotation away from the striatal lesion [47,48]. The findings coming from studies employing apomorphine-induced rotational behaviour as an outcome measure consistently indicated that in 6-OHDA PD rats, intrastriatal injections of BoNT led to a reduction of the anticlockwise rotational behaviour contralateral to the injected striatum [25,26,27,30,31,33,35,36,37,39,40]. Moreover, the BoNT-induced effects in 6-OHDA PD rats were more intense and prolonged when a second injection was administered at the same site after 6 months [30]. These effects were found to vary between BoNT subtypes, with BoNT-2A achieving the abolition of the apomorphine-induced rotational behaviour at a lower dosage than BoNT-1A [27]. Conversely, three studies reported that intrastriatal BoNT injections resulted in a significant increase in ipsilateral amphetamine-induced rotations [33,36,37]. Amphetamine acts as an indirect agonist of monoamines and administration to 6-OHDA lesioned rats causes a stronger delivery of dopamine to the contralateral striatum causing an ipsilateral clockwise rotation to the lesioned side. Although its application is simple, the amphetamine rotation test is a weak predictor of 6-OHDA-induced lesions and its interpretation may not be straightforward [62]. The persistence of this rotational behaviour after BoNT striatal injections may be due to the loss of catecholaminergic afferents in the 6-OHDA PD rat that is not influenced by BoNT and the further increase in this rotational behaviour after injection could be explained by changes in the basal ganglia circuits that may involve non-dopaminergic neurotransmitter systems [36]. 

In addition to drug-induced behaviours, several authors used validated motor tasks to test BoNT’s effects on motor function in animal models of PD, showing heterogeneous results. 

BoNT intrastriatal injections failed to influence spontaneous locomotor activity as well as forced motor activity [36,37]. Similarly, BoNT did not modulate lateralised sensori-motor integration [30,37]. Conversely, intrastriatal BoNT injections improved forelimb akinesia and equalisation of forelimb usage for six months after injection [31,36,39].

Only one study investigated the effects of BoNT injections in the striatum contralateral to the 6-OHDA-induced lesion [37]. The authors reported a transient increase in rotations induced by apomorphine, accompanied by a transient reduction in amphetamine-induced rotations 2 weeks after the injection. In addition, contralateral BoNT injections induced a significant improvement in forelimb use preference symmetry and forelimb akinesia and neglect contralateral to the 6-OHDA-induced lesion from 2 weeks to 9 months after the BoNT striatal injection [37].

Two studies evaluated the effects of administration of BoNT into the EPN, equivalent to human internal globus pallidus, on measures derived by a quantitative gait analysis in 6-OHDA PD rats [35,40]. In these studies, the assessment of static and dynamic gait parameters was obtained using the CatWalk apparatus, a video-based automated gait analysis system developed to evaluate footfall and gait changes in rodents. The BoNT injection into the EPN abolished the apomorphine-induced rotational behaviour, increased the gait speed and cadence, reduced gait speed variations, and improved dynamic gait parameters beginning 1 week after the injection, peaked 1 month later, and returned to basal values at 3 months [35,40].

Finally, our search identified one study in which a sample of a mouse model of PD was studied. In this study, Ham et al. found that BoNT striatal injections can improve forced motor activity, increase the latency to fall in the rotarod test, reduce bradykinesia (pole test), and improve gait parameters such as stride length and stance length in both 6-OHDA and MPTP PD mice [25].

Overall, the results coming from studies that have evaluated BoNT-induced effects on motor behaviour in PD animal models suggest its use as a potential therapeutic option in PD (see Table 3).

#### 3.1.3. Evidence for BoNT Effects on Non-Motor Behaviours

In our systematic review, we identified two studies that evaluated the effects of BoNT intracerebral injections on non-motor symptoms in PD animal models.

One study evaluated BoNT-induced effects on anxiety and depression in PD animal models. 

To evaluate the presence of behavioural manifestations of mood disorders in rats, several validated tests, including the open field test, the elevated plus maze test for anxiety-like behaviour, the forced swim test (FST), and the tail suspension test for depressive-like behaviour, were used (see Table 2). The authors observed that 6-OHDA PD rats did not show an increase in anxiety behaviours compared to naive rats, thereby preventing the authors from evaluating a potential effect of intrastriatal BoNT administration on these symptoms. Conversely, 6-OHDA PD rats had an increased depressive-like behaviour that significantly improved after BoNT treatment. In this paper, the authors failed to find a correlation between motor performance and depressive behaviour before and after the intrastriatal BoNT injections. This led them to conclude that the severity of the motor impairment might not influence improvements in affective symptoms in PD animal models [41].

Finally, a single study evaluated the olfactory performance changes in 6-OHDA PD rats after treatment with intrastriatal BoNT injections. Even though 6-OHDA rats had comparable olfactory performance with naive rats, the authors described an improvement in the execution of an orienting odour identification test in BoNT-treated compared to sham-treated 6-OHDA rats [34]. 

### 3.2. Evidence in Humans

In our review, we did not identify any clinical or neuroimaging studies providing indirect evidence of a central effect of BoNT in patients with PD. However, a recent neurophysiological study provided novel evidence on possible central mechanisms of BoNT in PD patients [42]. In a cohort of 12 “de-novo” and 7 “L-dopa-treated” tremulous PD patients, the authors used various transcranial magnetic stimulation (TMS) paradigms to evaluate intracortical inhibition/facilitation and sensory–motor integration before and 6 weeks after a BoNT injection in the forearm muscles. The TMS protocols were performed in both hemispheres before BoNT infiltration and 6 weeks after BoNT injection, extending over 42 weeks. Eight time points were considered for four cycles of BoNT injections. The inhibitory intracortical circuits were evaluated using a short intracortical inhibition (SICI) paradigm, in which a sub-threshold conditioning stimulus was followed by an above-threshold test stimulus with inter-stimulus intervals of 2 ms (SICI2) or 4 ms (SICI4). Additionally, prolonged intra-cortical inhibition (LICI) was assessed, where a supra-threshold conditioning stimulus with an interstimulus interval of 100 ms preceded the test stimulus. On the other hand, Short Afferent Inhibition (SAI) and Long Afferent Inhibition (LAI) paradigms were employed to assess sensory–motor integration involving the stimulation of a median nerve preceding the TMS pulses. This peripheral pulse inhibits the motor response from contralateral motor cortex stimulation, with specific interstimulus intervals of 23 ms for SAI and 200 ms for LAI [42,63]. 

The findings of this study demonstrated that intramuscular BoNT injections resulted in changes in cortical neurophysiological parameters. Specifically, de novo tremulous PD patients at baseline exhibited reduced SICI, LICI, and SAI on the tremulous/treated side compared to the non-tremulous side. However, the BoNT treatment led to an increase in LICI, SAI, and LAI and a reduction in ICF. By adopting a linear mixed model analytical approach, the authors found that the BoNT-induced changes in LICI, SICI, and LAI were significantly related to the changes in tremor severity, as assessed by a kinematic tremor analysis [42].

The evidence coming from this study might suggest that the efficacy of BoNT in the treatment of PD tremors is partially attributable to central mechanisms and to modulation of intracortical inhibitory circuits and sensory–motor integration mechanisms. 

## 4. Discussion

In this systematic review, we aimed to comprehensively evaluate the findings derived from studies conducted on animal models and human patients that focused on elucidating the potential central effects of BoNT in PD. Most of the evidence we reviewed arose from studies involving animal models of PD. At the same time, limited data were available from human studies, highlighting a gap in our current understanding and application of BoNT in the clinical management of PD. 

### 4.1. How Does BoNT Interact with PD Pathways? Evidence from Animal Models

The evidence coming from animal studies contributes to clarifying the multifaceted impact of BoNT on various neurotransmitter systems within the central nervous system. At the level of the cholinergic system, intrastriatal BoNT injections induce the expected blockage of Ach release as well as morphological changes in cholinergic neurons and a time-dependent modulation of cholinergic receptor density [26,29,46]. In the striatum, under normal physiological conditions, a complex and bidirectional interaction exists between the cholinergic and dopaminergic systems [64]. Multiple lines of evidence suggest that in PD, the loss of dopaminergic input leads to a reduced inhibition of tonically active cholinergic striatal interneurons [65,66]. This results in a state of relative cholinergic hyperactivation, which contributes in part to the motor symptoms of the disease [67]. Therefore, BoNT’s ability to modulate cholinergic transmission may represent one of the central mechanisms by which BoNT induces motor improvement in animal models. This hypothesis is, however, speculative, given that the animal studies did not investigate the potential relationship between BoNT-induced effects on the cholinergic system and motor and non-motor clinical improvement. 

Similarly, BoNT administration at the striatal level modulates compensatory mechanisms arising from dopaminergic denervation. Indeed, BoNT reduces the overexpression of dopaminergic receptors and normalises dopamine release levels [31,32,33]. The modulation of dopaminergic receptors seems clinically relevant given that two studies reported a correlation between the pattern of receptor expression and motor impairment in 6-OHDA PD rats [31,32,33]. The mechanism by which BoNT reduces dopamine receptor expression remains unknown, but cytotoxicity is ruled out, as the number of striatal neurons remains unchanged post-BoNT injection [26]. The regulation of dopaminergic receptor expression after BoNT injections may be due to the complex interplay between dopaminergic and cholinergic interneurons at the striatal level, as supported by observations in a PET study in monkeys [25]. 

In regard to glutamate, in our search, only the group of Tsang et al. targeted the EPN, i.e., the rodent equivalent of the GPi, and showed that BoNT can reduce glutamatergic transmission from the STN to EPN. Intriguingly, the authors observed that the chronological trajectory of BoNT-induced glutamatergic modulation was similar to that of BoNT-induced motor behavioural changes. The authors observed that, by blocking the glutamatergic influences from the STN, BoNT improved the gait parameters evaluated through the gait analysis [35]. Clinical and neurophysiological studies have described that dopaminergic denervation in PD induces an increased influence of STN efferents to the GPi and that the neuronal fibres that project from the STN to GPi are mainly glutamatergic [68,69,70]. This evidence is in contrast to what we observe in human PD patients treated with STN DBS, in which, the presence of axial symptoms, imbalance, and gait disturbances are considered a contraindication to implantation [71]. BoNT’s ability to modulate the abnormally increased glutamatergic transmission in PD animal models may contribute to BoNT’s clinical effects. 

Conversely, BoNT treatment does not appear to significantly influence the GABAergic, noradrenergic, and serotonergic systems in PD animal models, suggesting that its therapeutic effects might not be mediated through these systems.

### 4.2. Are BoNT’s Central Mechanisms Clinically Relevant in Animal Models of PD? 

A few authors evaluated the clinical effects of BoNT injections at the central nervous system level in animal models of PD. Drug-induced motor behaviours and validated motor tasks were used to evaluate BoNT-induced motor changes. Regarding BoNT’s effects on drug-induced motor behaviours, the most consistent finding is that BoNT injections into the striatum of 6-OHDA PD rats induces a reduction in apomorphine-induced rotations [26,27,30,31,33,36,37,39]. The apomorphine-induced rotation test is considered a good predictor of the effectiveness of 6-OHDA-induced lesions in PD animal models. After dopaminergic denervation, apomorphine, a dopamine receptor agonist, stimulates the supersensitive DR1 and DR2 of the dopamine-depleted hemisphere more than those in the contralateral hemisphere, causing an anticlockwise rotation away from the lesion site [47]. The abolishment of or reduction in this rotatory behaviour after BoNT treatment suggests the action of BoNT in counteracting the striatal compensation mechanisms for the dopaminergic denervation due to DR expression changes [36]. However, using the apomorphine rotation test in other animal models of PD has shown less reproducible results [72]. Antipova and Mann described an enhanced amphetamine-induced rotation behaviour in 6-OHDA PD rats treated with BoNT injections [33,36,37]. 

In addition to the study of drug-induced motor behaviours, the analysis of spontaneous behaviours of PD animal models after BoNT striatal injections has been carried out using different validated tests and showing variable results [25,30,31,35,36,37,39,40]. The observed variability, which significantly complicates the comparison of evidence across various research groups, arises from the utilisation of diverse animal models, variations in sample sizes, differences in methodologies, varying drug doses, distinct outcome measurement tests, and disparities in the latency between the intervention and measurement.

In terms of the effect of BoNT treatment on non-motor symptoms, only mood disorders and hyposmia have been explored so far. BoNT can induce a reduction in depression-like behaviours in 6-OHDA PD rats [41]. The improvement in depressive-like behaviour is independent of motor improvement after BoNT treatment, suggesting the presence of distinct pathophysiological mechanisms. These observations align with our current knowledge of non-motor symptoms in PD. It has been largely described that depression can precede the motor onset by up to months, is independent of the motor symptom severity, and has distinct pathophysiological mechanisms [73]. 

Focusing on hyposmia, Alberts et al. found that hemi-PD rats showed no olfactory deficits. BoNT striatal injections significantly improve olfactory performance and increase DR expression in the OB. A connectomics study demonstrated the presence of indirect connections between the striatum and the olfactory bulb, which could explain the modulation of DR expression in the OB after BoNT administration into the caudate–putamen complex [34]. These findings prompted the authors to posit that this influence could occur through anterograde and retrograde transportation of BoNT, a phenomenon previously described in the optic pathway of other animal models [74,75]. Nevertheless, even in the context of olfactory function, employing an animal model that mimics the symptoms of the disease without replicating the specific etiopathogenetic mechanism raises uncertainties about the extrapolation of these observations to human subjects with PD. 

### 4.3. Can BoNT Exert Central Effects in Patients with PD? 

Despite the predominance of animal model-based findings, only one study provided compelling evidence that BoNT, when intramuscularly injected, also exerts its action on the central nervous system and leads to significant time-dependent modifications in several neurophysiological measures of intracortical inhibitory circuits and sensory–motor integration in patients with PD [42]. In this study, the authors demonstrated that in PD patients with tremors, BoNT induces a reduction in tremor severity that may be related to the reorganisation of intra-cortical inhibitory activity, secondary to the action of BoNT on muscular afferent inputs and to an improvement in sensory–motor integration, as tested by the TMS parameters SAI and LAI. In line with this hypothesis, previous observations showed that an effective treatment for PD tremors is subthalamic DBS which also improves both SAI and LAI [76,77].

These preliminary findings not only highlight the potential of BoNT to modulate central neural pathways beyond its known peripheral effects, broadening our comprehension of its therapeutic impact in PD, but they also emphasise the need for further research to elucidate the central mechanisms of BoNT, aiming to refine and enhance the therapeutic strategies in the management of PD.

### 4.4. Limitations and Gaps of the Current Literature

Regarding the use of animal models to investigate BoNT’s effects on motor and non-motor symptoms, the translatability of evidence arising from animal models to humans is limited by several factors. First, the experimental design involves directly injecting BoNT into the central nervous system, while in clinical practice, BoNT is administered by intramuscular injections. Although retrograde and anterograde BoNT transportation from the muscle to the central nervous system has been hypothesised in animals, the experimental counterpart of these mechanisms in humans is still lacking [78,79]. Second, the animal studies investigating BoNT-induced behavioural changes generated a unilateral lesion of the striatum, failing to precisely replicate the etiopathogenetic and pathophysiological mechanisms of PD. Rather, they primarily capture the behavioural phenomenology resulting from a unilateral striatal lesion and may not comprehensively mirror the full spectrum of motor symptoms observed in human PD patients. Third, a significant limitation of animal studies assessing BoNT’s effects on mood disorders is represented by the challenge of identifying and assessing anxiety and depression in rats. To address this limit, the authors employed validated tasks capable of uncovering anxiety-like and depression-like behaviours in rats. Nevertheless, these tasks fall short of fully capturing the complex phenomenology of mood disorders associated with PD in humans. In addition, further investigations are needed to validate the existing evidence and to elucidate the impact of BoNT on other PD non-motor symptoms, which have not yet been examined in PD animal models but are currently under consideration for BoNT-based treatment. Fourth, the lack of investigation of possible clinical correlates mainly limits animal studies investigating BoNT effects on PD central pathways. Indeed, studies assessing BoNT’s effects on the cholinergic system did not explore the potential correlation between the molecular mechanisms and clinical changes. Conversely, the evidence on the clinical effects of BoNT’s action on the dopaminergic and glutamatergic systems is promising but still limited.

To date, the evidence in humans is represented by only one TMS study. Further neurophysiological and neuroimaging studies are, therefore, needed to clarify if central mechanisms are involved in the BoNT-induced clinical effects in patients with PD.

## 5. Conclusions

In conclusion, the evidence from PD animal models suggests that the intracerebral injection of BoNT leads to motor improvement by modulating the interactions between the cholinergic and dopaminergic systems at the striatal level. In humans, the evidence suggesting any potential central effects of BoNT is quite limited, with the only neurophysiological study we reviewed suggesting that the improvement in tremors following the peripheral administration of BoNT may be partly due to central mechanisms. The central mechanism through which BoNT may alleviate motor as well as non-motor symptoms of PD certainly requires further exploration and must be confirmed through additional neuroimaging, clinical, and neurophysiological studies in human subjects.

Elucidating BoNT’s central mechanism of action could provide novel insights into the pathophysiological mechanisms underpinning the various motor and non-motor symptoms of PD. Finally, this research could also improve PD management, enhancing the quality of life for patients and proving critical evidence for advancing personalised therapy by tailoring treatments to individual needs.

## Figures and Tables

**Table 1:** Studies included in our review divided by type of study in animal models (molecular evidence, evidence on motor behaviour and non-motor behaviour) and studies on human PD patients.

Animal Models				
1. Evidence for Molecular and Structural Changes				
1.1 BoNT Effects on Cholinergic System				
Author, Year	Subjects	Design	Methods	Main results
Wree et al., 2011 [26]	6-OHDA rats	Longitudinal (1 month)	Immunohistochemistry	BoNT induces axonal swelling in the infiltrated areas (BiVs). BiVs are reactive either for ChAT or for TH. BoNT does not exert toxic effects on cholinergic neurons.
Itakura et al., 2014 [27]	6-OHDA rats	Longitudinal (23 days)	Immunofluorescence analysis	BoNT-A2 increases cleaved SNAP-25 compared with that of the control group and the BoNT-A1 group.
Hawlitschka et al., 2017 [28]	C57BL/6 mice	Longitudinal (9 months)	Immunohistochemistry	BoNT does not exert toxic effect on cholinergic neurons. The density of BiVs shows a time-dependent decrease. No evidence of TH-ir BiVs in mice.
Mann et al., 2018 [29]	6-OHDA rats	Longitudinal (9 months)	Autoradiography	BoNT reduces interhemispheric differences for mAchRs and nAchRs in hemi-PD rats.
Hawlitschka et al., 2020 [30]	6-OHDA rats	Longitudinal (19 months)	Immunohistochemistry Stereological analysis	The density of striatal ChAT-ir neurons is higher in the injected striatum. BoNT does not exert toxic effects on cholinergic neurons. The density of ChAT-ir BiVs is higher in the twice-injected group.
1.2 BoNT Effects on Dopaminergic System				
Wedekind et al., 2018 [31]	6-OHDA rats	Longitudinal (5 months)	Histology Receptor autoradiography [11C]raclopride-PET/CT scans	BoNT-A reduces turning asymmetry. In 6-OHDA rats, there is a constant trend towards higher binding to striatal D2R. This trend tends to return to normal values after BoNT-A administration. BoNT-A induces a significant decrease in the D1R concentrations in contrast to sham-treated animals.
Mann et al., 2018 [32]	6-OHDA rats	Longitudinal (9 months)	Receptor autoradiography	BoNT reduces D2/D3 receptor density. Correlation between the intrastriatal D2/D3 reduction and the reduction in apomorphine-induced rotations after BoNT-A.
Mann et al., 2018 [33]	6-OHDA rats	Longitudinal (6 months)	[18F]fallypride-PET/CT scans	Hemi-PD rats have a constant increase in D2/D3R availability in the striatum. BoNT-A normalises the striatal availability of D2/D3R. Correlation between the BoNT-A effect on striatal D2/D3R and behavioural results in the apomorphine rotation test.
Ham et al., 2022 [25]	MPTP mice 6-OHDA mice	Longitudinal	Immunohistochemistry Western blot analysis ELISA (Dopamine; Ach) RT-PCR (TNF-α, IL-1β, IL-6)	BoNT-A: Ameliorates MPTP- and 6-OHDA-induced PD progression; Reduces acetylcholine release and levels of IL-1β, IL-6, and TNF-α as well as GFAP expression; Enhances dopamine release and tyrosine hydroxylase expression.
Alberts et al., 2022 [34]	6-OHDA rats	Longitudinal (6 months)	[18 F]fallypride-PET/CT Immunohistochemistry analysis fMRI	PET/CT and immunohistochemical data unchanged after formation of 6-OHDA-induced lesions. BoNT-A injection into the striatum induces an increase in the D2/D3R availability in the ipsilateral OB and concomitant improvement of olfactory performance.
1.3 BoNT Effects on Glutamatergic and GABAergic Systems				
Tsang et al., 2018 [35]	6-OHDA rats	Longitudinal (3 months)	Immunofluorescence	BoNT-A reduces synaptophysin and vGluT2 labelling.
1.4 BoNT Effects on Serotoninergic and Noradrenergic Systems				
Mann et al., 2018 [32]	6-OHDA rats	Longitudinal (9 months)	Receptor autoradiography	BoNT does not affect reduced alfa1, alfa2 and 5HT2a receptor density.
2. Evidence for BoNT Effects On Motor Behaviour				
Wree et al., 2011 [26]	6-OHDA rats	Longitudinal (12 months)	Drug-induced rotation test	BoNT-A abolishes apomorphine-induced rotations up to 6 months.
Antipova et al., 2013 [36]	6-OHDA rats	Longitudinal (12 months)	Drug-induced rotation test Forced motor test (accelerod test) Spontaneous motor test	BoNT-A abolishes apomorphine-induced rotations and enhances amphetamine-induced rotations. A dose of 2 ng BoNT-A tends to improve forelimb preference. BoNT-A does not influence spontaneous motor activity.
Itakura et al., 2014 [27]	6-OHDA rats	Longitudinal (23 days)	Drug-induced rotation test	BoNT-A2 ameliorates pathogenic rotation behaviour at a lower dosage than BoNT-A1.
Antipova et al., 2017 [37]	6-OHDA rats	Longitudinal (12 months)	Drug-induced rotation test Spontaneous motor test	Ipsilateral injection: ↓ Apomorphine-induced rotational behaviour; ↑ Amphetamine-induced turning rate. Contralateral injection: ↑ Apomorphine-induced turning rate; ↑ Performance in stepping test, cylinder test, OF test; ↑ Sensori-motor integration evaluated through the corridor test.
Wedekind et al., 2018 [31]	6-OHDA rats	Longitudinal (5 months)	Drug-induced rotation test Spontaneous motor test	BoNT-A reduces turning asymmetry. BoNT-A does not show a significant tendency to equalise forelimb usage.
Mann et al., 2018 [33]	6-OHDA rats	Longitudinal (6 months)	Drug-induced rotation test	BoNT-A reduces apomorphine-induced rotations.
Hawlitschka et al., 2018 [38]	6-OHDA rats	Longitudinal (12 months)	Drug-induced rotation test Spontaneous motor test	BoNT-A has no effect on corridor task and stepping test. BoNT-A reduces the apomorphine-induced rotations after both injections.
Tsang et al., 2018 [35]	6-OHDA rats	Longitudinal (3 months)	Gait analysis (CatWalk) Drug-induced rotation test	BoNT-A improves the rotational asymmetry and gait abnormalities of hemi-PD rats.
Antipova et al., 2019 [39]	6-OHDA rats	Longitudinal (12 months)	Drug-induced rotation test Spontaneous motor test	BoNT reduces initiation time in 6-OHDA rats.
Tsang et al., 2019 [40]	6-OHDA rats	Longitudinal (3 months)	Gait analysis (CatWalk)	BoNT-A has no effect on static gait parameters. BoNT-A increases dynamic gait parameters.
Ham et al., 2022 [25]	MPTP mice 6-OHDA mice	Longitudinal (3 weeks)	Motor behaviour testing (rotarod, pole test, and gait test)	BoNT induces improvement of motor behaviour test: Increases latency to fall; Decreases time to descend; Increases stride and stance length.
3. Evidence for BoNT Effects on Non-Motor Behaviours				
Antipova et al., 2021 [41]	6-OHDA rats	Longitudinal (1 months)	Drug-induced rotation test Spontaneous motor tests	Hemi-PD rats have increased depression-like behaviour compared with sham- or non-injected rats. Intrastriatal BoNT-A reduces depression-like behaviour in hemi-PD rats compared with the sham-injected control group.
Alberts et al., 2022 [34]	6-OHDA rats	Longitudinal (6 months)	Orienting odour identification test	Intrastriatal BoNT-A induces improvement of olfactory performance.
Human subjects				
Samotus et al., 2021 [42]	12 PD (de novo) 7 PD (L-Dopa) with tremor	Longitudinal (4 time points 6 weeks after BoNT injection)	Clinical scales Sensor-based tremor assessment Kinematics TMS (SICI, ICF, LICI, SAI, LAI)	At baseline on the tremulous side: ↓ SICI, LICI, SAI. BoNT-A treatment on the tremulous side: ↓ ICF; ↑ LICI, SAI; LAI. The changes in SICI, LICI, and LAI are significantly associated with changes in tremor severity.

**Abbreviations:** 5HT2a: 5-hydroxytryptamine receptor 2A, 6-OHDA: 6-hydroxydopamine, BiVs: BoNT induced Varicosities, BoNT: BotulinumToxin, ChAT: Choline acetyltransferase, D2R: Dopamine receptor, ICF: intracortical facilitation, IL: Interleukin, ir: interneuron, LAI: long afferent inhibition, LICI: long intra-cortical inhibition, mAchRs: Muscarinic acetylcholine receptors, MPTP: 1-methyl-4-phenyl-1,2,3,6-tetrahydropyridine, nAchRs: Nicotinic acetylcholine receptors, OB:olfactory bulb, OF: Open field test, PD: Parkinson Disease, SAI: short intracortical inhibition, SICI: short intracortical inhibition, TH: Tyrosine hydroxylase, TMS: Transcranial magnetic stimulation, TNF: Tumor Necrosis Factor, vGlut: vesicular glutamate transporter. **Legend:** ↑ increase, ↓ decrease.

**Table 2:** Validated tasks used to assess motor and non-motor behaviours in PD animal models.

Test	Function Explored	Paradigm	Reference(s)
Amphetamine-induced rotation	Drug-induced rotational behaviour to explore motor impairment after formation of 6-OHDA-induced lesion	Animals are injected with d-amphetamine sulphate (2.5 mg/kg, s.c.) and monitored for 60 min. Rotations are assessed using an automated, self-constructed rotometer system and defined as the number of complete 360° turns and registered as net differences between the two directions per minute.	Ungerstedt U et al., 1969 [47]
Apomorphine-induced rotation	Drug-induced rotational behaviour to explore motor impairment after formation of 6-OHDA-induced lesion	Animals are injected with apomorphine (0.25 mg/kg, s.c.), followed by registration of rotation for 40 min. Rotations are assessed using an automated, self-constructed rotometer system and defined as the number of complete 360° turns and registered as net differences between the two directions per minute.	Ungerstedt U et al., 1970 [48]
Open field test	Spontaneous locomotor activity	Rats are placed into a square arena (50 × 50 cm) with 50 cm high walls located inside of an isolation box. The running distance of the animals within 10 min is registered as a measurement of spontaneous locomotor activity.	Walsh et al., 1976 [49]; Basso DM et al., 1995 [50];
Cylinder test	Forelimb usage/preference	The use of the left and right forepaws during vertical exploration in a glass cylinder with a diameter of 20 cm is documented and analysed with a video camera system to count the initial contacts of the right or left paw and calculating the ratio of left to right forepaw use.	Schallert T et al., 2000 [51]
Rotarod/accelerod test	Forced motor activity	The kinematic analysis of forced motor activity is performed by computerised rotating rods starting at 4 rounds per minute (rpm) and accelerating to 40 rpm over a period of 5 min. The time spent on the rod before falling off and the maximum speed level reached are recorded.	Jones BJ et al., 1968a [52]; Jones BJ et al., 2011 [53];
Corridor test	Lateralised sensory–motor integration	Rats are placed for 5 minutes in a testing corridor in which the researcher have positioned bowls containing pellets in the right and the left side. The number of right side and left side retrievals are counted and the data are expressed as the percentage of left or right side retrievals over the total number of retrievals.	Döbrössy et al., 2007 [54]; Dowd et al., 2005 [55];
Stepping test	Forelimb akinesia	The rat is held by the investigator with one hand blocking both its hind limbs and the unrestrained forepaw touching the table. The rat is moved slowly sideways across the table and the number of adjusting steps of the respective unrestrained left or right forepaw are counted while moving in the forehand and backhand directions. Finally, the means of forehand and backhand steps of the left and right paws are calculated.	Olsson et al., 1995 [56]
Pole test	Bradykinesia	For the pole test, the time to turn and total time to place four paws on the base were measured after placing the mice at a fixed distance from the top of a metal rod (the pole).	Hwang et al., 2017 [57]
Elevated plus maze test	Anxiety-like behaviour	The rat is positioned in an elevated mace apparatus consistent of a central platform, an open arm, and a closed arm. During a 5 min test, the following are evaluated: time on the open arms, presence on open arms (% open time), and walking speed. All these parameters are inversely connected to anxiety.	Pellow et al., 1985 [58]
Forced swim test	Depressive-like behaviour	The rat is positioned in a forced swimming tank for 10 min and video recorded. During the task, the time spent struggling, time spent swimming, and time spent immobile are evaluated.	Porsolt et al., 1977 [59]
Tail suspension test	Depressive-like behaviour	Rats are slowly lifted grasping the base of the tail for a total of 60 s. The time (s) the rat spent immobile is considered a correlate of depression-like behaviour.	Chermat et al., 1986 [60]
Buried pellet test	Olfactory performance	The rat is put in a cage with 3 cm of clean bedding and one pellet buried 0.5 cm below in one corner of the cage or in the surface of the bedding. The rat is placed in the centre of the test cage and the latency time is measured until the rat uncovers the pellet and begins eating it.	Lehmkuhl et al., 2014 [61]

**Table 3:** Variability in motor behaviour testing after intrastriatal BoNT injection in PD animal models.

	Wree 2011 [26]	Antipova 2013 [36]	Itakura 2014 [27]	Antipova 2017 [37]		Wedekind 2018 [31]	Hawlitschka 2020 [30]	Mann 2018 [33]	Antipova 2019 [39]	Tsang 2018 [35]	Tsang 2019 [40]	Ham 2022 [25]
Animal model	6-OHDA PD rats	6-OHDA PD rats	6-OHDA PD rats	6-OHDA PD rats		6-OHDA PD rats	6-OHDA PD rats	6-OHDA PD rats	6-OHDA PD rats	6-OHDA PD rats	6-OHDA PD rats	6-OHDA and MPTp PD mice
Injection site	CPu	CPu	CPu	CPu		CPu	CPu	CPu	CPu	EPN	EPN	CPu
Injection side	ipsi	ipsi	ipsi	ipsi	contra	ipsi	ipsi	ipsi	ipsi	ipsi	ipsi	ipsi
Apomorphine-induced rotations	Abolished (3 months)	Abolished (4 weeks)	BoNTA2 reduced rotations with a lower dose than BontA1	Decreased (3 months)	Unchanged (2 weeks)	Reduced	Decreased (6 months) (more pronounced if repeated)	Decreased	Decreased	Abolished (1 week–1 month)	n.p.	n.p.
Amphetamine-induced rotations	n.p.	1 ng: No changes 2 ng: Enhanced (3 months)	n.p.	Increased (6 months)	Unchanged (2 weeks)	n.p.	n.p.	Increased	n.p.	n.p.	n.p.	n.p.
Gait tests	n.p.	n.p.	n.p.	n.p.	n.p.	n.p.	n.p.	n.p.	n.p.	n.p.	n.p.	Increased stride and stance length
CatWalk apparatus (gait impairment)	n.p.	n.p.	n.p.	n.p.	n.p.	n.p.	n.p.	n.p.	n.p.	Improvement in gait (1 week–1 month)	Improvement in dynamic gait parameters (1 week–1 month)	n.p.
Stepping test (forelimb akinesia)	n.p.	n.p.	n.p.	Unchanged	Improvement (up to 9 months)	n.p.	No improvement	n.p.	Initiation time (until 6 months)	n.p.	n.p.	n.p.
Corridor test (lateralised sensori-motor integration/neglect contralateral to 6-OHDA-induced lesion)	n.p.	n.p.	n.p.	Unchanged (6 months)	Improvement (up to 9 months)	n.p.	No improvement (6 months)	n.p.	n.p.	n.p.	n.p.	n.p.
Rotarod/accelerod test (forced motor activity)	n.p.	Unchanged	n.p.	n.p.	n.p.	n.p.	n.p.	n.p.	n.p.	n.p.	n.p.	Increased latency to fall
Cylinder test (forelimb usage/preference)	n.p.	Equalisation of left and right forepaw	n.p.	Unchanged	Improvement (2 weeks)	Reduction in forelimb use asymmetry	n.p.	n.p.	n.p.	n.p.	n.p.	n.p.
Open field test (spontaneous locomotor activity)	n.p.	Unchanged	n.p.	Unchanged	Unchanged	n.p.	n.p.	n.p.	n.p.	n.p.	n.p.	n.p.

CPu: caudate–putamen complex; EPN: entopeduncular nucleus; ipsi: ipsilateral to the lesioned striatum; contra: contralateral to the lesioned striatum; n.p.: not performed.

## Data Availability

No new data were created or analysed in this study. Data sharing is not applicable to this article.
